# Micelle-Forming Block Copolymers Tailored for Inhibition of P-gp-Mediated Multidrug Resistance: Structure to Activity Relationship

**DOI:** 10.3390/pharmaceutics11110579

**Published:** 2019-11-05

**Authors:** Alena Braunová, Martin Kaňa, Júlia Kudláčová, Libor Kostka, Jan Bouček, Jan Betka, Milada Šírová, Tomáš Etrych

**Affiliations:** 1 Institute of Macromolecular Chemistry, Czech Academy of Sciences, Heyrovského nám. 2, 162 06 Prague 6, Czech Republic; kudlacova@imc.cas.cz (J.K.); kostka@imc.cas.cz (L.K.); etrych@imc.cas.cz (T.E.); 2 Institute of Microbiology, Czech Academy of Sciences, Vídeňská 1083, 142 20 Prague 4, Czech Republic; martin.kana@biomed.cas.cz (M.K.); sirova@biomed.cas.cz (M.Š.); 3 Department of Otorhinolaryngology and Head and Neck Surgery, First faculty of Medicine, Charles University and University Hospital Motol, V Úvalu 84, 150 06 Prague 5, Czech Republic; Jan.Boucek@fnmotol.cz (J.B.); jan.betka@fnmotol.cz (J.B.)

**Keywords:** polymer therapeutics, multidrug resistance, micelles, P-glycoprotein, solid tumors, block copolymers

## Abstract

Multidrug resistance (MDR) is often caused by the overexpression of efflux pumps, such as ABC transporters, in particular, P-glycoprotein (P-gp). Here, we investigate the di- and tri- block amphiphilic polymer systems based on polypropylene glycol (PPO) and copolymers of (*N*-(2-hydroxypropyl)methacrylamide) (PHPMA) as potential macromolecular inhibitors of P-gp, and concurrently, carriers of drugs, passively targeting solid tumors by the enhanced permeability and retention (EPR) effect. Interestingly, there were significant differences between the effects of di- and tri- block polymer-based micelles, with the former being significantly more thermodynamically stable and showing much higher P-gp inhibition ability. The presence of Boc-protected hydrazide groups or the Boc-deprotection method did not affect the physico-chemical or biological properties of the block copolymers. Moreover, diblock polymer micelles could be loaded with free PPO containing 5–40 wt % of free PPO, which showed increased P-gp inhibition in comparison to the unloaded micelles. Loaded polymer micelles containing more than 20 wt % free PPO showed a significant increase in toxicity; thus, loaded diblock polymer micelles containing 5–15 wt % free PPO are potential candidates for in vitro and in vivo application as potent MDR inhibitors and drug carriers.

## 1. Introduction

Malignant neoplasms are life-threatening and devastating human diseases. To improve the anti-tumor efficiency of low molecular weight (LMW) anticancer drugs, various drug delivery systems and polymer-based prodrugs have been designed and investigated in preclinical and clinical trials [1]. However, multidrug resistance (MDR) [2,3], either intrinsic or occurring as a result of chemotherapy, is often caused by the overexpression of ATP-dependent efflux pump P-glycoprotein (P-gp) in tumor cells [4]. The role of this important transmembrane protein in the organism is to transport a wide range of substrates (over 300 compounds) [4,5,6] including antineoplastic agents and other drugs, across extra- and intra- cellular membranes. Thus, the intracellular concentration of the drug is reduced to subtherapeutic levels due to P-gp activity before the drug can fulfil its mission within the tumor cells, thereby reducing the efficacy of chemotherapy in cancer treatment [4]. Currently, there is an effort to overcome MDR by the co-administration of P-gp inhibitors with anticancer drugs; such LMW inhibitors of P-gp include verapamil, cyclosporine A, ritonavir, or reversin [4]. However, these compounds have several disadvantages, e.g., short circulation in the blood and non-selective biodistribution, which lead to serious side effects on healthy cells, e.g., liver hepatocytes with increased P-gp level [4]. These drawbacks can be overcome either by covalent or non-covalent attachment of the inhibitors to high molecular weight (HMW) polymer carriers, alone or in combination with anticancer drugs [7,8,9]. Another method is the use of HMW P-gp inhibitors such as poly(propylene glycol) (PPO) copolymers [10,11,12,13], whose activity is well-known from the literature [14,15]. Pluronics, triblock amphiphilic copolymers of poly(ethylene glycol) (PEG) and PPO, have been extensively studied as polymer carriers of non-covalently attached hydrophobic drugs (doxorubicin, taxols) as well as micellar HMW polymer inhibitors.

Micelles are extensively studied drug delivery systems (DDS) because of their ability to prolong the circulation of the drug in bloodstream, improve the pharmacokinetics of the carried drug, and protect the drug against biotransformation inside the body. The formation of micelles from individual polymer chains (unimers) in suitable, mostly aqueous media is thermodynamically driven, in particular, by concentration and temperature, and this process is reversible [16]. Unimers are usually amphiphilic block copolymers with molecular weights below the renal threshold. Immediately after their self-assembly into micelles, a rapid increase in molecular weight and size is observed. This phenomenon has been successfully utilized for superior accumulation of these systems in solid tumors by enhanced passive accumulation due to the enhanced permeability and retention (EPR) effect [17,18,19,20,21]. The micelles are composed of a hydrophobic core and hydrophilic corona or shell. The hydrophobic core can serve as a reservoir of the hydrophobic drug or other hydrophobic active molecules, while the hydrophilic corona should sterically prevent interactions of the hydrophobic cargo with body liquids and/or blood proteins. The hydrophobic parts of amphiphilic copolymers are often polymers and copolymers, such as PPO or other systems [16,22,23,24,25,26,27] e.g., poly(amino acid)s, biodegradable poly(esters) like poly(ε-caprolactone), poly(glycolic acid), poly(d-lactic acid) and poly(d,l-lactic acid) or their copolymers, and polyphospholipids/long chain fatty acids. In contrast, PEG is mainly used as a hydrophilic block in micelles [16,22,23], with the drug bound to the polymer carrier covalently by chemical conjugation (e.g., by biodegradable hydrazone bond), or non-covalently entrapped using physical interaction, solubilization, or polyionic complexation [16]. Some micellar systems with various hydrophobic drugs (doxorubicin, paclitaxel, cisplatin, epirubicin, etc.) entrapped in the micellar core based on poly(amino acid)s like poly(l-glutamic) or poly(α,β-aspartic) acid have been tested in clinical trials [22,23].

Recently, we described a new micellar amphiphilic block copolymer based on hydrophilic *N*-(2-hydroxypropyl)methacrylamide copolymer (PHPMA) and PPO as the hydrophobic part. Moreover, the micellar conjugate with anthracycline antibiotic doxorubicin (Dox) covalently attached via pH-sensitive hydrazone bond was also synthesized and tested [21]. The PHPMA-PPO-Dox copolymer micelles proved their excellent therapeutic potential as a passively targeted DDS in vivo. Notably, the PHPMA-PPO copolymer micelles without the drug exhibited effective chemosensitization in human and murine cancer cell lines overexpressing P-gp, but without the concurrent toxicity.

The present work is a pilot study focusing on the detailed structure–function relationship and the improvement of the MDR inhibition of PHPMA-PPO copolymer micelles by the loading of free PPO into the micellar core. The P-gp inhibitory activity of PPO itself was much higher than the inhibitory effect of PPO bound in the PHPMA-PPO copolymer. Here, PPO-based P-gp inhibition is sterically hindered due to the hydrophilic shell based on PHPMA chains. The influence of functional groups, the methods of their removal, and different content of non-covalently incorporated PPO on the physico-chemical properties (hydrodynamic size, stability in solution, and the ability of self-assembly in aqueous media) and biological behavior in vitro (P-gp inhibitory activity and cytotoxicity) was evaluated. Based on these results, we concluded that PPO loaded PHPMA-PPO copolymer micelles may be excellent therapeutic DDS for pH-sensitive drug delivery, and simultaneously, P-gp inhibitors. These loaded micelles sensitize in situ the chemoresistant tumor cells towards the chemotherapy; thus, they can overcome the frequent problem of MDR in chemotherapy of solid tumors.

## 2. Materials and Methods

### 2.1. Chemicals

The following chemicals were purchased from Sigma-Aldrich (Prague, Czech republic): 1-Aminopropan-2-ol; methacryloyl chloride; 6-aminohexanoic acid; 2,2’-azobisisobutyronitrile (AIBN), methyl 6-aminohexanoate hydrochloride; tert-butyl carbazate; trifluoroacetic acid (TFA); *N,N′*-dicyclohexylcarbodiimide (DCC); *N,N′*-dimethylacetamide (DMA); dimethyl sulfoxide (DMSO); triisopropylsilane (TIS); 2,4,6-trinitrobenzenesulfonic acid solution (1M in H_2_O); thiazoline-2-thiol; 2,2´-azoisobutyronitrile (AIBN); 4,4′-azobis(4-cyanopentanoic acid) (ACVA); 4-cyano-4-(ethylthiocarbonothioylthio)pentanoic acid, *N*-(3-dimethylaminopropyl)-*N′*-ethylcarbodiimide hydrochloride (EDC.HCl), *N,N´*-dicyclohexylcarbodiimide (DCC), 4-dimethylaminopyridine (DMAP), hydrazine monohydrate, Nile Red (Bioreagent ≥ 98%; NR), 2-thiazoline-2-thiol, and poly(propylene glycol) bis(2-aminopropyl ether) (*M*_w_ = 4000 g/mol; PPO). All other chemicals and solvents were of analytical grade. The solvents were dried and purified by conventional procedures.

### 2.2. Synthesis of Monomers and Initiator

*N*-(2-hydroxypropyl)methacrylamide (HPMA; comonomer 1) was synthesized according to the literature [28]. M.p. 69–70 °C; Elemental analysis: calculated/found: C 58.72/58.98%, H 9.15/9.18%, N 9.78/9.82%. 1-(*tert*-Butoxycarbonyl)-2-(6-methacrylamidohexanoyl)hydrazine (Ma-Ah-NHNH-Boc; comonomer 2) was prepared as described previously [29]. M.p. 114–116 °C; Elemental analysis: calculated/found: C 57.49/58.26%; H 8.68/8.95%; N 13.41/13.25%. The initiator 2-(1-cyano-1-methyl-4-oxo-4-(2-thioxo-thiazolidin-3-yl)-butylazo)-2-methyl-5-oxo-5-(2-thioxothiazolidin-3-yl)-pentanenitrile (ABIC-TT) was prepared according to a previously described method [30].

### 2.3. Synthesis of Thiazolidine-2-Thione Functional CTA 2-Cyano-5-oxo-5-(2-thioxo-1,3-thiazolidin-3-yl)pentan-2-yl Ethyl Carbonotrithioate (CTA-TT)

The synthesis of CTA-TT containing trithiocarbonyl groups was undertaken according to a recently described method [31]. Briefly, 4-cyano-4-(ethylthiocarbonothioylthio)pentanoic acid (1.51 g, 5.72 mmol) and 2-thiazoline-2-thiol (0.71 g, 6.0 mmol) were dissolved in 15 mL of dichloromethane (DCM), EDC·HCl (1.50 g, 7.80 mmol) was added to the solution, followed by the addition of a catalytic amount of DMAP. The reaction mixture was stirred at laboratory temperature for 2 h, then extracted with distilled water (2 × 20 mL); the organic layer was dried with anhydrous Na_2_SO_4_ before the DCM was evaporated. The oily residue was diluted with a mixture of hexane:ethyl acetate 1:1 (30 mL) and purified by silica gel chromatography using a mixture of hexane:ethyl acetate (1:1). The eluent was evaporated, and the yellow oily product was crystallized in a refrigerator, yielding 1.33 g (64%). HPLC gave a single peak at 305 nm with a retention time of 11.7 min. ESI−MS: *m*/*z* = 365.08 (M-H)+; ^1^H NMR (600 MHz, CDCl_3_) 4.59 (t, 2H, NCH_2_CH_2_), 3.60–3.66 (m, 2H, CH_3_CH_2_S), 3.34 (t, 2H, CH_2_CH_2_S), 3.34 (t, 2H, CH_2_CH_2_CO), 2.49 (t, 2H, C(CH_3_)CH_2_CH_2_), 1.89 (s, 3H, (CH_3_)C(CN)), 1.36 (t, 3H, CH_3_CH_2_).

### 2.4. Synthesis of Hydrophilic Polymer Blocks A1 and A2 Based on PHPMA Copolymers

Semitelechelic hydrophilic block A1, poly(HPMA-*co*-Ma-Ah-NHNH-Boc)-TT, with terminal TT groups and protected hydrazide groups along the polymer chain, was synthesized by RAFT polymerization with molar ratio ABIC-TT/CTA-TT/comonomers 1/2/80. The ratio of comonomers HPMA/Ma-Ah-NHNHBoc was 92/8 mol %. HPMA (5 g; 34.9 mmol) was dissolved in *tert*-butanol (46.1 mL), and Ma-Ah-NHNHBoc (950 mg; 3.04 mmol), ABIC-TT (229 mg; 0.474 mmol), and CTA-TT (346 mg; 0.948 mmol) were dissolved in DMSO (8.1 mL). The polymerization mixture was bubbled with argon for 15 min. The copolymerization was performed in a sealed ampule at 70 °C for 16 h. The product was isolated by precipitation with an acetone/diethylether (1:1) mixture, centrifuged, filtered, and dried. The reactive ω-end trithiocarbonate groups (TTc) were removed with 2,2´-azobisisobutyronitrile (AIBN), as previously described [32]. The conversion was 77% (4.6 g).

The hydrophilic copolymer A2 with a similar structure but a different molecular weight to block A1 was prepared analogously as described above, with a molar ratio ABIC-TT/CTA-TT/comonomers (1) and (2) of 1/2/65.

The semitelechelic copolymers were characterized by size exclusion chromatography (SEC). The functionality of main chain terminated TT groups was determined as the ratio *M*_n_**/***M*_n_,_TT_, where *M*_n,TT_ is the number-average molecular weight calculated from the TT group content. The content of NHNH_2_ groups was determined spectrophotometrically, and the hydrodynamic diameter (*D*_h_) was determined by dynamic light scattering (DLS). The characterization data of both copolymers are listed in Table 1, and details of the methods used for the characterization are described in Section 2.10 (physico-chemical characterization).

### 2.5. Purification of Hydrophobic Polymer Block B Based on PPO before the Reaction

Before diblock formation, commercial poly(propylene glycol) bis(2-aminopropyl ether) (NH_2_-PPO-NH_2_; M 4000 g/mol) was purified by column chromatography using Sephadex LH20 in methanol with UV detection (230 nm), RI, and TLC in chloroform/methanol (5/1), with the visualization solution consisting of NH_4_SCN (3.4 g), Co(NO_3_)_2_ (0.56 g), and water (20 mL). The content of the terminal amino groups was determined spectrophotometrically, as described earlier [21].

### 2.6. Synthesis of Polymer Carriers: Diblock and Triblock Micellar Copolymers

#### 2.6.1. Boc-Protected Diblock Copolymer PHPMA-b-PPO P1 and P2

Diblock copolymer P1, PHPMA-b-PPO, with a non-degradable amide bond between hydrophilic A1 and hydrophobic B block was synthesized as described previously [21]. Briefly, hydrophilic polymer A1 (263.2 mg; 0.036 mmol TT groups), as well as hydrophobic polymer B (145.4 mg; 0.036 mmol PPO), were dissolved separately in a molar ratio 1:1 (TT of copolymer A1: copolymer B) in dimethylacetamide (DMA). The solution of A1 was slowly added to the solution of B while stirring at room temperature, and the reaction mixture was reacted overnight. The product was then purified by column chromatography (Sephadex LH20, methanol, UV at 230 nm, and TLC detection), and the polymer solution was evaporated under vacuum to yield the Boc-protected diblock copolymer P1. The rest of the original unreacted PPO in copolymer P1 was then removed from the crude product (before lyophilization) by leaching with dichloromethane (DCM), which was poured off, followed with lyophilization from water to yield Boc-protected diblock copolymer P2 purified from free PPO. The characteristics of copolymers P1 and P2 are presented in Table 2.

#### 2.6.2. Diblock Copolymers P3 and P4, PHPMA-b-PPO, with Deprotected Hydrazide Groups in Distilled Water at Elevated Temperature and Pressure

The tert-butyloxycarbonyl (Boc) groups protecting hydrazide groups located along the PHPMA backbone of the copolymer P1 and P2 were removed according to the literature, using hydrolysis at high temperature [31,33]. First, the P1 or P2 diblock copolymer was dissolved in water in an ampule (concentration approximately 5% vol.) and bubbled with argon for 5 min. The ampule was sealed and heated at 100 °C in an oil thermostat for 30 min, then cooled, opened, and the final copolymer P3 or P4 free of unbound PPO was obtained by lyophilization without any other purification, with a yield of 97% for both polymers. The characteristics of copolymers P3 and P4 are listed in Table 2.

#### 2.6.3. Diblock Copolymer P5, PHPMA-b-PPO, with a Deprotected Hydrazide Group by the Mixture of TFA/TIS/Water

Alternatively, the Boc groups were removed from the copolymer P1 as described earlier [21] using acid hydrolysis with a mixture of 95% TFA/2.5% triisopropylsilane (TIS)/2.5% H_2_O, followed by evaporation, neutralization, desalination using a PD10 column (Sephadex G-25), and final lyophilization, to yield diblock copolymer P5. The characteristics of copolymer P5 are summarized in Table 2.

#### 2.6.4. Triblock Copolymer P6, PHPMA-b-PPO-b-PHPMA

The triblock copolymer P6 was prepared by the reaction of A2 with B in an approximate ratio of 2:1, using a slight excess of the hydrophilic block A2 as follows: hydrophilic copolymer A2 (396 mg; 0.064 mmol TT groups) and hydrophobic polymer B (107 mg; 0.054 mmol NH_2_ groups) were separately dissolved in DMA. The solution of B was slowly added to the A2 solution while stirring, and the reaction mixture was reacted overnight. Then, the product was purified and isolated under the same conditions as the Boc-protected diblock copolymer P2. After the evaporation of methanol, the rest of the unreacted PPO was removed using DCM leaching. Thereafter, the triblock product was separated from the unreacted PHPMA copolymer A2 and diblock copolymer by column chromatography (Sephacryl TM S300 high resolution, GE Healthcare; phosphate-buffered saline with pH 7.4) and lyophilized. The triblock polymer fraction was collected, desalinated by column chromatography (Sephadex G25; distilled water), deprotected at 100 °C using water in a sealed ampule, and lyophilized analogously, as in the case of the diblock copolymer P4. The final triblock copolymer P6 was characterized by SEC, field flow fractionation (FFF), NMR, and DLS; the characteristics are shown in Table 2.

### 2.7. Loading of Free PPO into the Diblock Polymer Micelles

The amphiphilic diblock copolymer P4 and corresponding amount of PPO (see Table 3) were dissolved together in 0.5 mL MeOH and the solution was evaporated under vacuum to dryness. The residue was redissolved in 0.5 mL methanol, the same volume of distilled water was added, and methanol was very slowly evaporated under vacuum. The residue solution was diluted with distilled water to the final concentration of resulting micelles of 10 mg mL^−1^, and the product was isolated by lyophilization, yielding loaded micelles P7–P14. The micelles loaded with free PPO were characterized by SEC, FFF (*M*_n_ of micelles; ratio of micelles: unimers), DLS (size of particles; time-dependent stability of particles at 37 °C; critical micellar concentration (CMC) of micelles), Maldi TOF (presence of free PPO), and NMR (content of total amount PPO: free PPO in micelles) as shown in Table 4.

### 2.8. Time-Dependent Micellar Stability

Samples were dissolved in PBS (concentration 1 mg mL^−1^) and filtered using 0.20 µm PVDF filter. After 10 min, the polymer solution was filtered using a 0.45 µm filter (PVDF). The micellar solution was equilibrated at 37 °C for 10 min and measured using DLS at 0, 24, 48, and 72 h after equilibration.

### 2.9. Critical Micellar Concentration

CMC was determined by fluorescent dye Nile Red (NR) according to the literature [34,35]. Samples were dissolved in 10 mM PBS (concentration 1 mg/mL) and diluted to a final concentration 10^−6^ mg mL^−1^. A solution of NR (final concentration of NR in samples ~1 × 10^−6^ mol L^−1^) in ethanol (5 vol % in the final sample) was added. After 30 min, the fluorescence was measured at λ_ex_/λ_em_ ~530/610 nm on a Synergy H^1^ hybrid Reader instrument (96-well flat bottom black microplate). The CMC was determined as the intersection of two straight lines drawn through points at low and high concentrations from the graph of dependence of fluorescent intensity on the negative logarithm of the sample concentration.

### 2.10. Physico-Chemical Characterisation

Monomers and CTA-TT were characterized by HPLC Shimadzu system (Kyoto, Japan) with a reverse-phase column Chromolith high resolution RP-18e, 100 × 4.6 mm (Merck, Darmstadt, Germany) equipped with UV/VIS photodiode array detector. The gradient elution was 5−95% of acetonitrile for 15 min at a flow rate of 1.0 mL min^−1^. The weight-average molecular weights (*M*_w_), number-average molecular weights (*M*_n_), and dispersities (*Ð*) of the prepared polymers, and copolymers were determined using a HPLC Shimadzu system equipped with a UV detector, an Optilab^®^rEX differential refractometer, and a multi-angle light scattering HELLEOS II™ (Wyatt Technology, Santa Barbara, CA, USA) detector and size-exclusion chromatography TSK gel Super SW 3000 (Tosoh Bioscience, Griesheim (Darmstadt), Germany) and/or Zenix C300 (Sepax Technologies, Newark, DE, USA) columns. The *M*_w_, *M*_n_, and *Đ* were calculated using the Astra 7 software (Wyatt Technology, Santa Barbara, CA, USA). The Optilab^®^-rEX detector enables the direct determination of the refractive increment (dn/dc) of the polymers; the solvent refractive index provides 100% recovery of the injected sample from the column. The refractive index increment dn/dc (for PHPMA copolymers ~0.167 mL g^−1^; for PPO ~0.130 mL g^−1^; for PHPMA-PPO copolymers ~0.150 mL g^−1^) was used for the calculation. A 20% sodium acetate buffer (150 mM, pH 6.5): 80% methanol (*v*/*v*) as a mobile phase for the analysis was used with a flow rate of 0.3 mL min^−1^. The amount of TT reactive groups in the polymer precursor was determined using UV/VIS spectrophotometry with the molar extinction coefficient ε_302nm_ = 10,500 L mol^−1^ cm^−1^ in methanol (Specord 205, Analytik Jena, Jena, Thuringia, Germany). The functionality of TT groups was calculated as *M*_n,T_/*M*_n_, i.e., the molecular weight determined for the polymer containing 1 mol of main chain-end TT group divided by molecular weight determined by SEC. The molecular weight of the formed micelles and the ratio between micellar and unimer form of copolymers were determined by FFF (Eclipse 3+ modul) equipped with DLS (DAWN 8+; Wyatt Technology Corp., Santa Barbara, CA, USA), UV and RI (Optilab rEX;Wyatt Technology Corp., Santa Barbara, CA, USA) detection using a regenerated cellulose membrane (10 kDa) for the analysis and a mixture of water/NaN_3_ as the mobile phase. The concentration of the sample was 3 mg mL^−1^. The content of hydrazide groups after deprotection was determined spectrophotometrically using a TNBSA assay in 0.1 M Na_2_B_4_O_7_·H_2_O with pH 9.3 (ε_500_ = 17,200 L mol^−1^ cm^−1^). The total PPO content (bound and free; wt %) in the di- or tri- block copolymers P1–P14, respectively, was determined using a ^1^H-NMR (Bruker Avance III 600 spectrometer, Bruker BioSpin GmbH, Rheinstetten, Germany, 600.2 MHz, 296 K) from the ratio HPMA:Ma-AhNHNH_2_:PO. The integral intensities from the ^1^H NMR spectrum in MeOD were compared: δ 3.94 (br), ^1^H (CH–OH) from HPMA and δ 3.69 (br), 1H (CH–CH_3_) from PO. The presence of unbound PPO was determined by MALDI TOF in dimethylformamide (DMF) using calibration against an internal standard. The hydrodynamic diameters (*D*_h_) of the di- or tri- block polymer micelles in a phosphate-buffered saline (1 mg mL^−1^; pH 7.4) were performed on a Nano-ZS instrument (ZEN3600, Malvern, UK), as were the long-term stability of the micelles in aqueous media. The micelles were incubated for 72 h in PBS (pH 7.4) at 37 °C to determine their stability in an aqueous environment. To ensure dust-free cells prior to DLS experiments, all solutions were filtered with 0.22 µm PVDF filter for PBS buffer and 0.45 µm PVDF filter for micellar samples. The intensity of the scattered light was detected at angle *θ* = 173° using a laser with a wavelength of 632.8 nm. To evaluate the dynamic light scattering data, the DTS (Nano) program was used. The values were a mean of at least five independent measurements. Values were not extrapolated to zero concentration.

### 2.11. Cell Lines

The murine monocytic leukaemia P388 cell line and its Dox resistant subline P388/MDR were kindly gifted by Professor I. Lefkovits (Basel, Switzerland). The cells were cultured under standard conditions (37 °C, 5% CO_2_ atmosphere) in RPMI 1640 medium (Sigma-Aldrich) supplemented with 10% heat inactivated fetal calf serum (FCS; Gibco), 1 mM sodium pyruvate, 0.1 mM non-essential amino acids, and antibiotics (penicillin/streptomycin, Sigma-Aldrich). The P388/MDR cells were kept under selective pressure in the presence of 750 ng mL^−1^ of Dox to maintain the MDR phenotype, and 24 h before experimental use, were transferred to a drug-free medium. All cell lines were free of mycoplasma (MycoAlert Mycoplasma detection Kit, Lonza, Basel, Switzerland).

### 2.12. Calcein Efflux Assay

The ability of the diblock copolymers to inhibit P-gp was determined using a modification of the calcein efflux assay, as described previously [36]. The P388/MDR cells and the cells of P388 parental cell line were seeded at a concentration of 1 × 10^5^ per well in 150 µL of culture medium (96-well flat bottom plate, Thermo Fisher Scientific) and incubated with titrated concentrations of the diblock copolymer (ten 1:2 serial dilutions in 50 µL of culture medium) for 24 h at 37 °C. As a positive control, 10 µM cyclosporine A (CsA) was added for the last 30 min of cultivation. Subsequently, Calcein AM (Invitrogen) was added at final concentration of 0.2 µM, and the cells were incubated for 30 min at 37 °C protected from light. Next, the cells were washed twice and resuspended in 100 µL of ice-cold FACS buffer (PBS supplemented with 2% FCS and 2 mmol EDTA). The intensity of calcein fluorescence was determined using a BD LSRII flow cytometer. The dead cells were identified and gated using Hoechst 33258 (Sigma-Aldrich). In each sample, 20,000 living cells were counted. An unpaired Student’s t-test was employed to analyze the differences in the intensity of the calcein fluorescence. Experiments were performed in triplicate; representative diagrams are shown ±SD.

## 3. Results and Discussion

### 3.1. Synthesis of Hydrophilic Blocks A1, A2 and Unloaded Polymers P1–P6

**Scheme 1:**
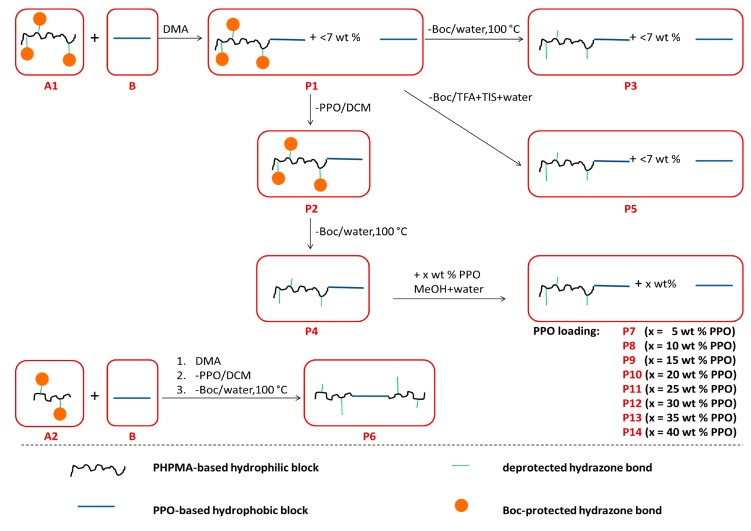
Scheme of the syntheses of copolymers P1–P14.

A series of various amphiphilic diblock or triblock copolymers were synthesized based on PHPMA and PPO, as described below in Scheme 1. Their physico-chemical properties, i.e., molar weights in different environment, ability to self-assembly into the micellar structures, CMC, hydrodynamic size, or long-term stability, with crucial biological properties in vitro such as P-gp inhibition ability in MDR tumor cells and toxicity, were compared.

To achieve similar molar weights of final di- or tri- block amphiphilic copolymers, two PHPMA copolymers A1 and A2 were synthesized with identical structures but different molar weights, i.e., being significantly higher for A1, the precursor of all diblock copolymers. Both hydrophilic polymer blocks were synthesized by controlled RAFT radical polymerization of HPMA and Ma-Ah-NHNHBoc comonomer using CTA containing trithiocarbonate (TTc) and TT groups and TT-functionalized azoinitiator. The reaction was followed by postpolymerization removal of TTc groups with 2,2´-azobisisobutyronitrile at 70 °C [32]. The type of polymerization, i.e., controlled RAFT polymerization, was chosen because it provides a narrow distribution of molar weights of resulting copolymers, in our case up to *Ð* ~1.06. The weight-average molar weights (*M*_w_) of synthesized hydrophilic PHPMA blocks determined by SEC in organic mobile phase were 7600 (*M*_n_ ≈ 7200) for A1 and 5100 (*M*_n_ ≈ 5000) for A2. TTc groups were removed prior to the use of polymers in further reactions to exclude possible interaction of TTc groups in biological experiments. After removing TTc groups, Boc-protected diblock or triblock amphiphilic copolymers were prepared by aminolytic reaction of the terminal reactive TT groups of semitelechelic hydrophilic polymers A1 or A2, with one or both terminal amino groups of PPO, respectively.

**Figure 1:**
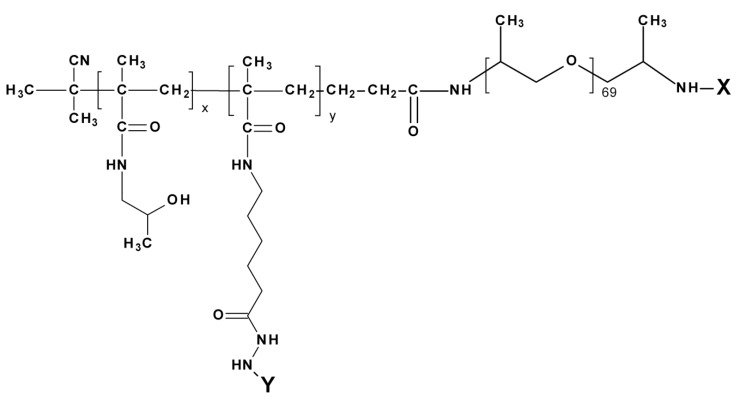
The schematic structure of amphiphilic block copolymers based on PHPMA and PPO: (X) = H, diblock copolymer; (X) = PHPMA, triblock copolymer; (Y) = Boc, Boc-protected copolymer; (Y) = H, deprotected copolymer with free NHNH_2_ groups.

The obtained amphiphilic block copolymers, diblock P1 and P2 and triblock P6, had similar molar weights (*M*_n_), showing their structure and amphiphilic balance similarity, i.e., about 13,000 for diblock copolymers P1 and P2 and approximately 13,400 for triblock P6. The schematic structures of the block copolymers based on PHPMA and PPO are shown in Figure 1. After the reaction, a small amount (less than 7%) of unreacted PPO was determined in both the diblock and the triblock samples, and the free PPO was removed by leaching with DCM to obtain the P2 and P6 polymers. For a higher yield of the triblock polymer carrier, a slight excess (about 15%) of polymer A2 was used in the reaction. Due to the use of a non-stoichiometric ratio of PHPMA to PPO and lower TT functionality of hydrophilic polymer A2, the triblock copolymer was free of all impurities, unreacted PPO and PHPMA, and unintended diblock. First, unreacted PPO was successfully eliminated from the di- and tri- block copolymers by leaching with DCM, in which the block copolymers are, due to insolubility of PHPMA block, poorly soluble in contrast with the free PPO. The final diblock P2 and triblock P6 polymers contained less than 3% of unreacted PPO after purification. Similarly, diblocks and unreacted PHPMA were removed from the triblock copolymer by gel chromatography using UV and RI detection to obtain entirely pure triblock copolymer P6 for subsequent studies.

All copolymers except P1 and P2 contained free hydrazide groups along the hydrophilic block chain. As described before [21], these functional groups could be used advantageously for the attachment of drugs or another biologically-active compounds via pH-sensitive hydrazone bonds. This shows pH-responsive behavior, as it is rapidly hydrolyzed in intracellular environments with lower pHs (lysosome, endosome), and is stable in the bloodstream with a pH of 7.4 [21]. Deprotection of the hydrazide groups in copolymers was carried out by two different methods to make a comparison in terms of amphiphilic copolymer synthesis. Usually, strong acid-based solutions, such as a mixture of 95% trifluoroacetic acid (TFA) with 2.5% triisopropylsilane (TIS) and 2.5% water, are used for the deprotection of Boc-protected amino or hydrazide groups. However, this method often results in an increase in polymer dispersity due to polymer crosslinking [31]. Based on the literature data [31,33], we also applied hydrazide group deprotection based on 30 min incubation in water at 100 °C in the preparation of the P3, P4, and P6 polymers. Compared to the TFA method, deprotection in boiling water does not require any other chemical or purification steps, like neutralization and desalination, because only gaseous side reaction products (isobutylene and CO_2_) are produced. Thus, the deprotection was followed by lyophilization only. There was no significant effect of either deprotection method on the physico-chemical properties of polymers P3 and P5 in comparison to the default polymer P1 without deprotection, indicating that there was no difference when both methods were applied. Analogously, no significant change in characteristics was found in deprotected P4 and Boc-protected P2 copolymers, both being purified from unreacted PPO. To sum up, there were no significant changes observed in the physico-chemical characteristics of all the diblock copolymers, either after the removal of Boc groups or following the purification of diblocks from free PPO.

Generally, it is accepted that the self-assembly of amphiphilic copolymers into micelles is a thermodynamic and reversible process which is driven entropically. Conventional core-shell micelles are formed when the weight of the hydrophilic block is similar or slightly greater than the hydrophobic part [16,27]. In this study, this was fulfilled in both cases, i.e., diblock and triblock copolymers, because the weight of the hydrophilic PHPMA block is about 1.7-times larger than that of the hydrophobic PPO block. Moreover, it was proved previously [21] that PHPMA-PPO micelles are self-assembled into micelles spontaneously by direct dissolution in aqueous solution (water, PBS buffer etc.) above CMC. Thus, the tumor uptake of these copolymers should be enhanced because of passive targeting into the solid tumor due to the well-known EPR effect [17,19].

All synthesized block copolymers based on PHPMA-PPO were linear amphiphilic copolymers with a molecular weight below the renal filtration threshold. Thus, these compounds should be excreted from the body by glomerular filtration after fulfilling their role as drug carriers after the disassembly of their micellar structures into single chains as a consequence of dilution in the circulation in blood or tissues. Their self-assembling behavior into the micelles gives rise to a dramatic increase their apparent molar weights, i.e., more than fifty times when compared to their unimer molar weights, also significantly increasing their hydrodynamic size when compared to those of hydrophilic blocks A1 and A2 (see Table 1 and Table 2).

**Figure 2:**
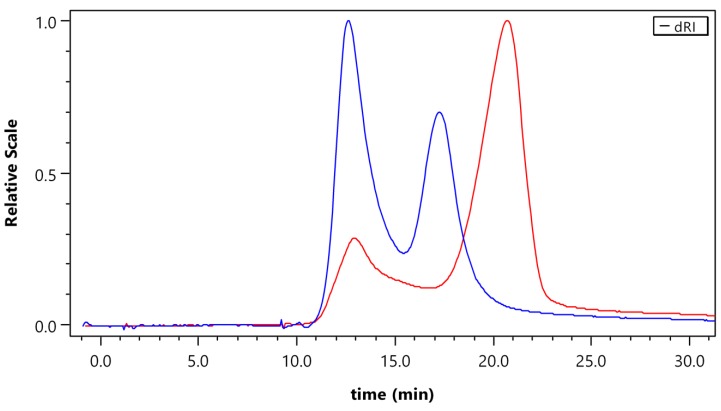
Chromatograms of molar weights of diblock P4 (red curve) and triblock P6 (blue curve) copolymers determined by FFF (3 mg mL^−1^; mobile phase: water/NaN_3_; 10 kDa regenerated cellulose).

In aqueous media, the molar weights of the resulting micelles were monitored using the FFF technique, in addition to determining the ratio between micellar and unimer fractions. As an example, the FFF chromatograms for deprotected diblock P4 and triblock copolymer P6, respectively, are shown in the Figure 2. For the triblock copolymer P6, we observed that in an aqueous environment, more than half of the P6 copolymer is in the form of unimers, 55% (*M*_n_ 25,000):45% (*M*_n_ 370,000), showing a low level of thermodynamic capability of P6 to aggregate into micelles. In contrast, the ratio micelles to unimers in the case of diblock copolymer P4 (23% of unimer, *M*_n_ 17,000:77% of micelles, *M*_n_ 697,000) was much more significant for the self-assembled micellar structures. Thus, the self-assembling ability of diblock copolymers is significantly higher than that in the triblock copolymer P6, regardless of their similar PPO content and molar weight. We hypothesize that the diblock construct forms thermodynamically more stable micellar structures, as the whole PPO chain is involved in the hydrophobic interaction between diblock copolymers. Conversely, the triblock copolymer should introduce the PPO chain into the core as a loop, leaving both PHPMA terminal chains outside the core in hydrophilic shell. The loop-like core structure is more prone to the disassembly than the core based on the rod-like structure of the diblock self-assembly; therefore, the amphiphilic diblock based micellar self-assembly is much more potent to form thermodynamically-stable micelles.

### 3.2. Size of the Particles (D_h_) and Long-Term Stability of Non-Loaded Polymers P1–P6

As described earlier [37], the size of the particles is one of most important factors influencing the circulation times and organ distribution of the micellar DDS. In general, particles with diameters smaller than 200 nm are less susceptible to reticuloendothelial system (RES) clearance, and those with a diameter below 50 nm are less accumulated in the liver, spleen, and lungs [37,38]. Here, the hydrodynamic diameters (*D*_h_) of all the prepared copolymers were determined in a PBS buffer mimicking the pH of the bloodstream using DLS. The sizes of all the studied P1–P6 micelles were in the range of 20 to 30 nm (see Table 2). Based on this knowledge, and on biocompatible shells based on PHPMA chains showing no soft and hard corona forming in contact to plasma proteins [39], we can expect that the micelles should not be taken up by RES during circulation in the bloodstream [21]. We observed a small difference in the sizes of the micelles formed from polymers P1–P6, with polymers P2, P4, and P6 freed from unreacted PPO having slightly smaller diameters compared to the non-purified polymers. Thus, the potential of free PPO to stay incorporated in the core of the micelle and increase the diameter by core enlargement was shown. The difference between P2 and P4 diblock and P6 triblock polymer micelles, all freed from PPO, was not statistically significant, indicating that the micelles formed by di- and tri- block polymers are similar in size.

**Figure 3:**
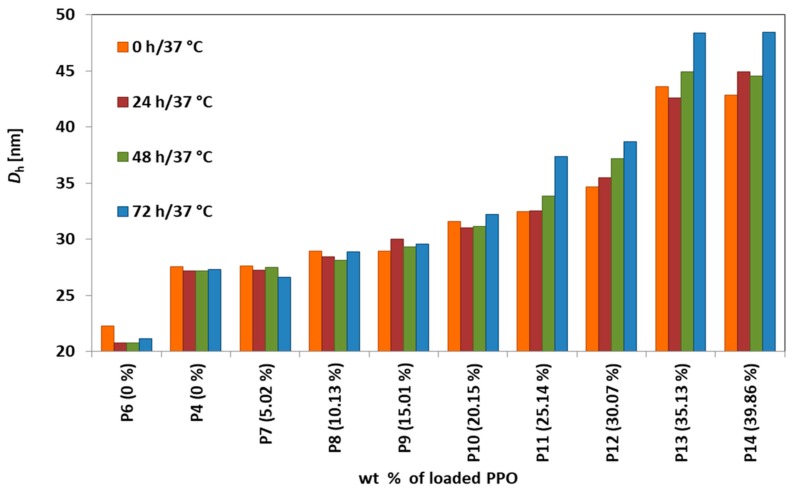
Size dependence of micelles P4, P6–P14 on the time of incubation, copolymer structure, and content of loaded PPO.

To verify the stability of the micelles based on diblock P4 and triblock P6 in an aqueous environment mimicking the bloodstream, the size of the self-assembled particles expressed as *D*_h_ was monitored and determined in PBS buffer by DLS measurement for 3 days. Both the studied polymers diblock P4 and triblock P6 micelles exhibited excellent stability during incubation (see Figure 3). This indicates that these micelles should be stable during circulation in the bloodstream, and that they will be disassembled after the drop of concentration at the final destination, i.e., the tumor tissue.

### 3.3. CMC of Non-Loaded Polymers P2–P6

**Figure 4:**
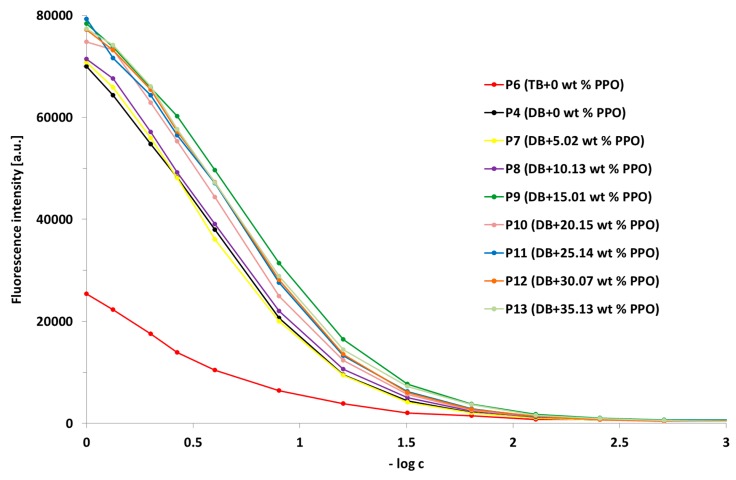
Dependence of fluorescent intensity on the concentration of the triblock and diblock micelles; fluorescent intensity of fluorescent dye Nile Red of diblock copolymers determined after 30 min at λ_ex_/λ_em_ = 530/610 nm in PBS buffer at 37 °C. The CMC was determined as the intersection of two straight lines drawn through points at low and high concentration from the graph of dependence of fluorescence intensity on negative logarithm of sample concentration.

The hydrodynamic stability of micelles formed from amphiphilic copolymers in solution depends on concentration. Below the CMC, the micelles disintegrate to single polymer chains (unimers), excludable from the body by renal filtration. The micellar form, in contrast, ensures their long-term circulation and accessibility for the solid tumor accumulation. NR labelled copolymers P4 and P6 were used for CMC determination. In a hydrophilic environment, NR has no fluorescence, but the fluorescence is detectable in a hydrophobic environment, i.e., in the micellar hydrophobic core. The data of the diblock and triblock are shown in the Figure 4, and the resulting CMC values of the polymers are listed in Table 2.

The CMC values of diblock P2–P5 and triblock copolymer P6 micelles differed significantly, with the triblock copolymer P6 being less stable under conditions mimicking the bloodstream than its diblock analogues (see Figure 4 and Table 2). This result corresponds well with the less favorable value of unimer-to-micelle ratio and slightly lower *D*_h_ of P6, determined by FFF and DLS, respectively, when compared to all diblock copolymers. Even though the molar weights of diblock P2–P5 and triblock P6 unimers were very similar, the data determined by FFF showed that apparent molar weights of the diblock and triblock copolymers micelles in aqueous solution (see Table 2) differed significantly (see Figure 2). Allen et al. [37] stated that the degree of steric stabilization depends on the properties of a micelle corona forming the hydrophilic block, in this case, PHPMA copolymer. We hypothesize that the triblock polymer structure with two shorter polymer chains significantly affects the ability of PPO to form hydrophobic interactions within the core of the micelles. Thus, the rod-like structure of the diblock polymer is preferable for the micellar structure, and leads to significantly more stabilized micelles. Here, we found 2.3-fold lower CMC for diblock P4 copolymer in comparison with the CMC value in P6, supporting the hypothesis that the diblock copolymers P2–P5 should be more thermodynamically stable than their triblock analogue P6. Finally, we can conclude that, due to the stability of micelles, the diblock polymer carrier is an interesting candidate for further investigation.

### 3.4. Diblock Copolymer Micelles Loaded with PPO, Samples P7–P14

Generally, loading into the micellar core is utilized for the solubilization of water-insoluble hydrophobic drugs and their safe transport to the site of action, as well as their protection against blood proteins and other components [16,27]. Similarly, we employed the micelle core solubilization technique for the encapsulation of free PPO as a potential highly effective P-gp inhibitor. We investigated in detail how the biological behavior of micellar copolymers is influenced by the presence of various amounts of free PPO, which we assume is a better P-gp inhibitor than PPO bound to the PHPMA in diblock copolymer. To ensure the effect of added free PPO, the original diblock copolymer was first freed of PPO by leaching with DCM.

It has been demonstrated [16,37,40] that the most important factor related to drug solubilization capacity of a polymer micelle is the compatibility between the drug and the micellar core-forming hydrophobic block. Here, we decided to incorporate the same PPO into the micellar core, thus anticipating the maximal compatibility between the PPO and the hydrophobic core formed by the same polymer. The diblock micellar copolymer P4 was successfully loaded with free PPO by slow solvent evaporation to yield loaded amphiphilic diblock based micelles P7–P14. The physico-chemical parameters of the loaded polymers P7–P14, such as the molar weights of micelles determined by FFF, hydrodynamic sizes of particles *D*_h_ or CMC values, are presented in Table 4.

**Figure 5:**
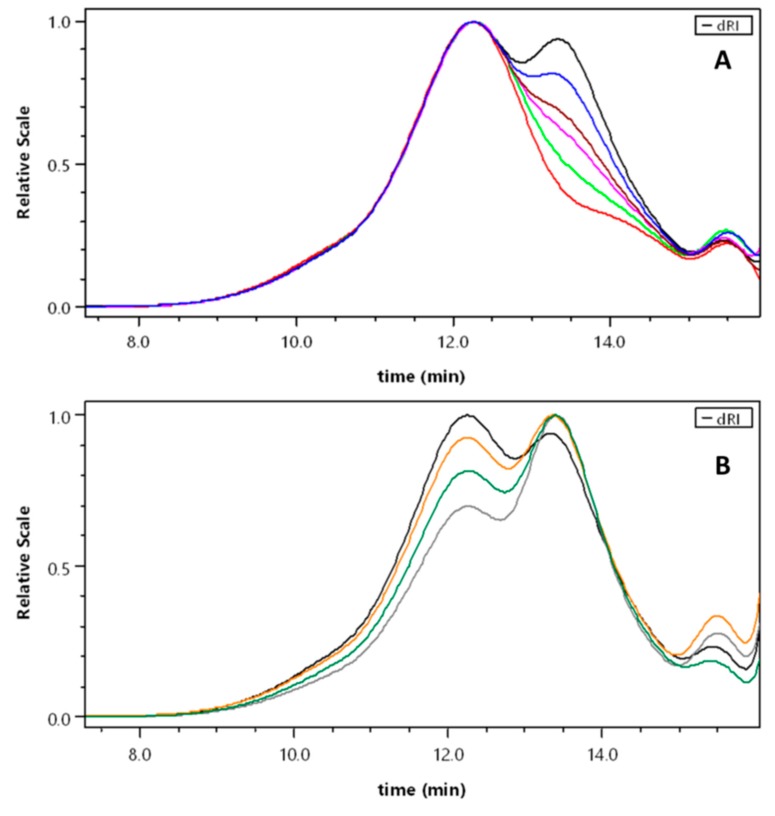
Loading of free PPO into micellar purified copolymer P4 (SEC, 80% MeOH/20% acetate buffer, pH 6.5; column TSK 3000 Super SWXL)—SEC chromatograms: (**A**) 0 (red curve), 5 (light green curve), 10 (pink curve), 15 (brown curve), 20 (blue curve), 25 (black curve) wt % of loaded PPO; (**B**) 25 (black curve), 30 (orange curve), 35 (green curve), 40 (grey curve) wt % of loaded PPO.

By measuring the unimers in the organic phase by SEC, we observed an increased amount of free PPO (peak maximum at 13.5 min) in the samples, showing successful PPO loading into the diblock **P4** sample; see Figure 5.

FFF analysis revealed that the molar weights of the loaded polymers ranged between 570,000 and 760,000 g/mol, with small differences between the ratio of unimers and micelles (from 70 to 80% of micelles). The highest apparent molar weight and micelle portion peaked in P9 copolymer micelle loaded with 15 wt % of PPO. The DLS measurements showed the increase in size of micelles with increasing free PPO content (see Table 4). *D*_h_ increased from 25.4 nm in non-loaded P4 to 42 nm in P14 with 40 wt % loaded PPO (see Figure 3). Taken together, the loading of 20 and more wt % of free PPO increases significantly the hydrodynamic size of the micelles. Hypothetically, the hydrophobic core in these samples is expanded by the significant number of stretched free PPO chains leading to the size increase. We have not observed significant differences in CMC in all the loaded copolymers. The highest stability was found for the P9, CMC value 0.0339 g L^−1^, proving its very good micellar stability in an aqueous media. Analogously, as shown for the unloaded micellar polymers P1–P6, we also observed good long-term stability of PPO loaded micelles P7–P14 during their incubation in the PBS buffer for 3 days (see Figure 3). Polymer micelles P7–P10 with lower PPO loading did not change the size in solution at all. In the case of PPO loaded diblock micelles P11–P14 with higher PPO loading, a small gradual increase in the size of nanoparticles (*D*_h_) during the incubation time was observed, but this size change was not significant. In addition, these highly loaded micellar systems, P11–P14 with 25–40 wt % of free PPO, are not applicable either in vitro or in vivo due to their cytotoxicity (see Section 3.5.2).

In summary, the increasing loading of PPO into the PHPMA-PPO micelles leads to the slight increase in the hydrodynamic size of the micelles in the solution. There is an almost negligible effect on the CMC of the prepared micelles, showing that all the unloaded, as well as loaded, micelles are stable in solution at very low concentrations. However, loaded micelles with more than 20 wt % PPO showed instability during long-term incubation. Thus, the loading of PPO in the range of 5–20 wt % is optimal for micellar systems with high stability and the appropriate physico-chemical properties.

### 3.5. Biological Activity

#### 3.5.1. The Influence of the Detailed Structure of the Copolymer Carrier on the P-gp Inhibitory Capacity

**Figure 6:**
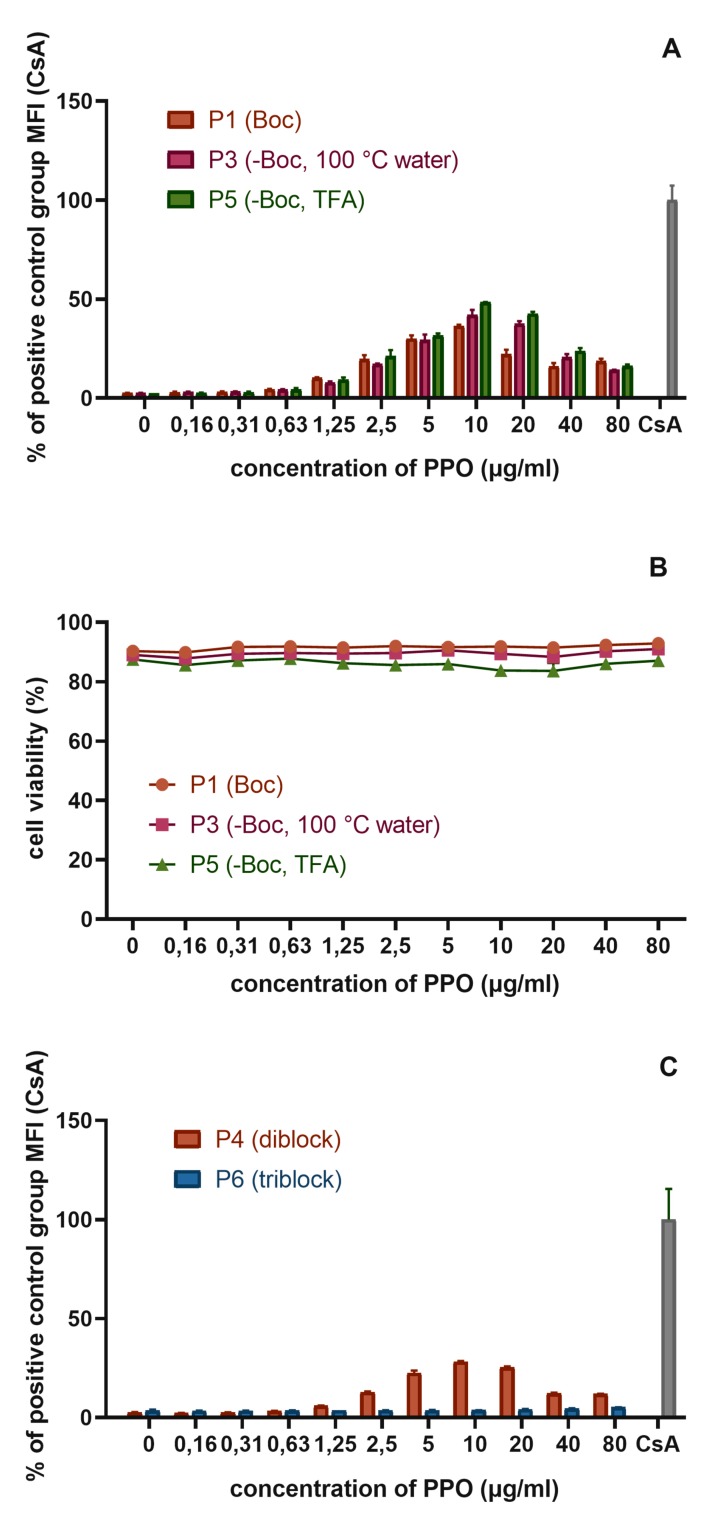
The inhibitory activity of block copolymer carriers in P388/MDR cells determined by calcein efflux assay. (**A**) The influence of Boc-deprotection on P-gp inhibition in P388/MDR cells after 24 h of cultivation (5% CO_2_, 37 °C) with titrated concentrations of block copolymers containing indicated amount of PPO; 10 µM cyclosporine A as the positive control; (**B**) The effect of micelle-forming copolymers on viability of P388/MDR cells determined using Hoechst 33258. Cell viability after 24 h of cultivation with Boc-protected and or Boc-deprotected copolymers; (**C**) The ability of Boc-deprotected diblock P4 and triblock P6 copolymers to inhibit P-gp function.

The ability of the PHPMA-PPO diblock copolymer to inhibit the function of P-gp transporters has already been described [21]. However, seemingly insignificant changes in the diblock copolymer synthesis or structure may affect its functionality. In vitro analysis of the P-gp inhibitory activity of the Boc-deprotected copolymers in the calcein assay showed an insignificant increase in the inhibitory capacity of the Boc-deprotected copolymers P3 and P5 containing free –NHNH_2_ groups, when compared to Boc-protected copolymer P1 (Figure 6A). Furthermore, the method used for the Boc-deprotection did not affect the P-gp inhibition efficiency, since both methods produced copolymers with equal activity in the calcein assay. The tested concentrations of the block copolymer carriers did not show any toxicity in P388/MDR cells (Figure 6B). Thus, the deprotection of the functional hydrazide groups distributed along the polymer backbone using either distilled water at 100 °C or a strong acid mixture (TFA/TIS/water) yields no difference of Boc-deprotection in the terms of the resulting P-gp inhibitory activity. The functionalization of the PHPMA polymer chain with hydrazide groups was previously shown to affect the copolymer kinetics in the body, namely blood clearance and tissue content, but the ability of the conjugate to accumulate in the tumor was not compromised [41]. The presence of hydrazide groups, speculatively, may affect the ability of the micellar block copolymer to interact with glycoproteins, namely N-glycans. Hydrazide functionalization of (bio)materials is being explored in order to enhance the affinity for glycopeptides [42]. Protein glycosylation, and in particular N-linked glycosylation, is prevalent in proteins destined for extracellular environments [43]. The P-gp is a heavily glycosylated membrane protein; glycosylation occurs on three N-linked recognition sites in the first extracellular loop. Though the contribution of the oligosaccharides to the structure and function of P-gp remains unclear, the sugar moieties are purported to play a role in plasma membrane expression and efficient function of P-gp. Moreover, it is also not known how the diblock micellar copolymer interacts with the P-gp molecule. The insignificant trend to the increased P-gp inhibitory activity observed in the Boc-deprotected copolymer seems consistent with these data. However, one should be aware that the hydrazide content is relatively small, and moreover, that some of the groups could be buried in the micellar structure, and thus be inaccessible. Therefore, the moderate effect of the hydrazide groups present in the micellar structure probably could not be considered significant for its overall therapeutic efficacy.

Next, we studied the influence of the structure of block copolymers on their biological activity in vitro. The Boc-deprotected triblock copolymer P6 and the Boc-deprotected diblock copolymer P4 with similar molar weights were compared for their capacity to inhibit P-gp function. Surprisingly, we did not observe any P-gp inhibition in P388/MDR cells treated with the triblock copolymer P6 (Figure 6C). Although we used analogous protocols for the synthesis of di- and tri- block copolymers, and their molar weights were similar, the difference in the structure resulted in very different behavior in aqueous solutions, and virtually deleted the biological activity of the triblock copolymer.

#### 3.5.2. The Effect of PPO Loading to the Micelles on P-gp Inhibition

**Figure 7:**
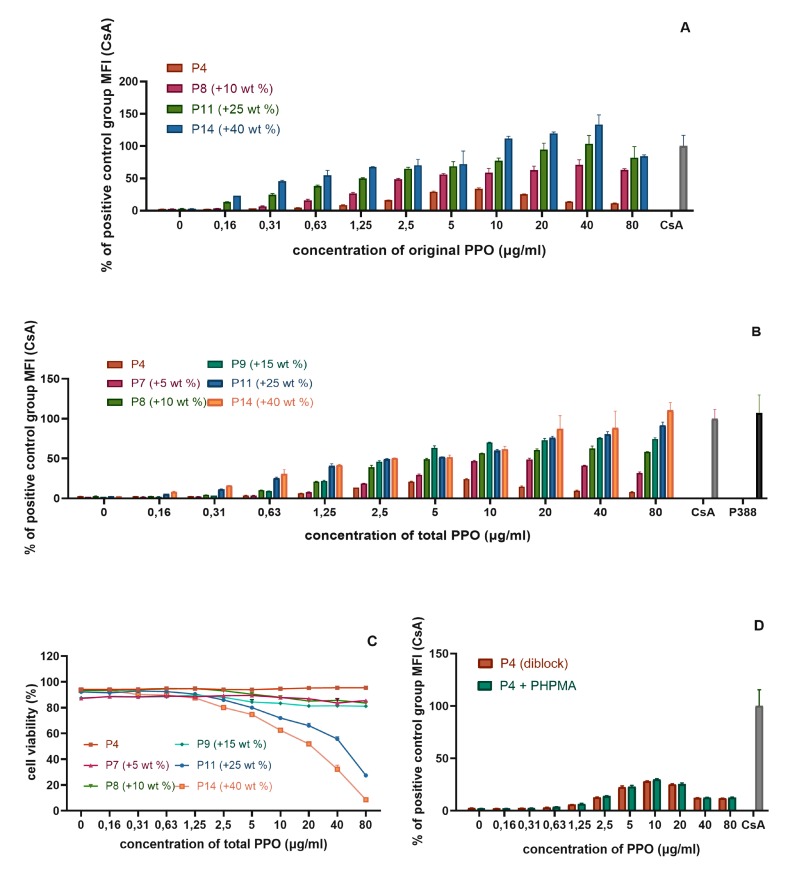
The P-gp inhibitory activity of PHPMA-PPO copolymers with different proportion of free PPO. The P388/MDR cells were cultivated for 24 h with titrated concentrations of copolymer samples and their P-gp activity determined by calcein assay; 10 µM cyclosporine A and sensitive P388 cells with calcein as the positive control (**A**) Samples resulting from adding various wt % of free PPO to the original sample P4, in which the total PPO content was determined by NMR. The *x*-axis indicates concentrations of PPO before loading of the free PPO, amount of the added free PPO as indicated in the legend; (**B**) Inhibitory activity of a series of copolymers (P4, P7, P8, P9, P11, and P14) with various ratios of PHPMA-PPO to the loaded free PPO. The *x*-axis indicates total content of PPO in the samples, which is the sum of the starting PPO in the original sample and the added free PPO; (**C**) Viability of P388/MDR cells cultivated with titrated concentrations of copolymer samples with various ratio of PHPMA-PPO to the loaded free PPO as shown in Figure 7B. Viable cells (Hoechst 33258-negative) were determined by flow cytometry. (**D**) Inhibitory activity of the mixture of PHPMA-PPO copolymer and added free PHPMA in a molar ratio of 1:1.

The loading of the additional free PPO to the micelles formed from diblock P4 increased their inhibitory activity in a dose-dependent manner (Figure 7A). In order to assess the extent to which the free PPO participates in the inhibition of P-gp, we repeated the testing of the series of samples with increasing ratio of free (loaded) PPO to the PHPMA-PPO copolymer P4 but with equal molar concentration of the total (sum of the starting and the loaded) PPO (Figure 7B). Apparently, the inhibitory activity increased with increasing the proportion of the loaded free PPO content, irrespective of the same total PPO amount. Even the loading of 5 wt % of PPO in P7 increased the inhibitory activity of the copolymer 2.5-fold when compared to the unloaded copolymer P4 at the same molar concentrations of total PPO (Figure 7B). This effect may be attributed to the higher P-gp inhibitory activity of free PPO in comparison, with the P-gp inhibition executed by PPO chemically conjugated to PHPMA copolymer. Thus, the difference between the P-gp inhibitory activity of the free and bound PPO is probably significant. However, this cannot be directly demonstrated, since the free PPO cannot be delivered other than as part of the diblock PHPMA-PPO micelle.

**Figure A1:**
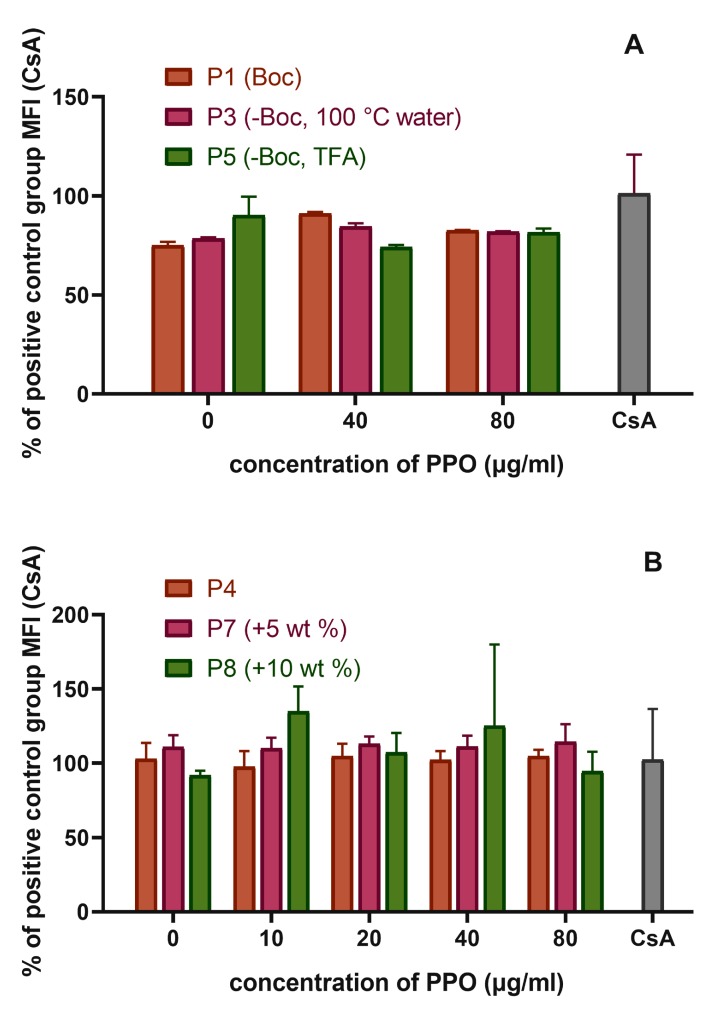
The activity of block copolymer carriers in sensitive P388 cells determined by calcein efflux assay. (**A**) The effect of Boc-protected or -deprotected copolymers on P-gp inhibition after 24 h of; 10 µM cyclosporine A as the positive control. (**B**) The effect of PHPMA-PPO copolymers with loaded free PPO.

Nevertheless, the loading of free PPO to the micelles also negatively affected the viability of the cells (Figure 7C), whereas the copolymer P9 with 15 wt % of loaded free PPO did not noticeably influence cell viability within the concentration range tested; the higher PPO loading resulted in considerably increased toxicity of the samples. Thus, the free PPO loading of micelles based on PHPMA-PPO must fall within a certain range of the amount of free PPO incorporated into hydrophobic core of these micelles due to the toxicity of the micelles with PPO loading higher than 15 wt %, as detected in vitro by calcein assay. Furthermore, we performed calcein efflux assay in P388 sensitive cell line treated with the tested compounds. However, as these cells do not express the MDR phenotype, we did not observe any increase in calcein accumulation in the tested concentrations; see Figure A1.

Contrary to the hydrophobic PPO block, increasing the PHPMA content in the resulting micelle by adding additional free linear PHPMA polymer to the diblock copolymer did not affect the inhibitory activity of the tested samples (Figure 7D) or the cell viability (data not shown).

## 4. Conclusions

A series of the polymer micelles based on diblock or triblock amphiphilic PHPMA-PPO copolymers was successfully prepared, and selected micelles were loaded precisely with an increasing amount of free PPO. The shape of the di- or tri- block copolymers substantially influenced the thermodynamic stability of the block copolymers and their P-gp inhibitory activity, with the diblock polymer micelles showing significantly enhanced capabilities of forming stable micelles in aqueous solution when compared to the triblock polymer micelles. The PHPMA-PPO diblock polymers formed stable micelles with suitable hydrodynamic sizes of around 30 nm, and with sufficient stability in solution. Interestingly, the change in the polymer structure from di- to tri- block copolymers caused a strong decrease in the P-gp inhibitory ability, showing the value of the diblock structure for P-gp inhibition. The structure of the diblock polymer micelles enabled loading up to 40 wt % of free PPO into the micelle core, leading to a slight increase in hydrodynamic size with increased loading of free PPO. Nevertheless, diblock micelles loaded with more than 20 wt % free PPO showed a significant increase in the cytotoxicity of the micellar carrier. Thus, loading from 5 to 15 wt % of free PPO is appropriate for drug delivery and P-gp application. Moreover, the presence of free PPO incorporated into the micelles increased the ability of the copolymer to inhibit the P-gp function in vitro to a much higher extent compared to that of the unloaded micelles. In conclusion, micellar amphiphilic block copolymers based on PHPMA and PPO loaded with free PPO in the hydrophobic core are potential advantageous drug carriers that passively target solid tumors due to the EPR effect, serving as highly potent HMW inhibitors of P-gp mediated MDR. Thus, the described micellar copolymer carriers conjugated with chemotherapy drugs may give rise to an effective means of treatment of MDR tumors, for which traditional chemotherapy is not satisfactory.

## Figures and Tables

**Table 1:** Characterization of semitelechelic hydrophilic block A1 and A2.

Sample Code	*M*_n_.10^3^ (g/mol)	*Ð*	Functionality of TT *^a^*	Content of NHNH_2_ (mol%)	*D*_h_, 37 °C (nm)
**A1**	7.2	1.06	0.99	6.0	6.1
**A2**	5.0	1.02	0.81	4.7	4.5

^a^ Functionality of TT determined as ratio *M*_n_/*M*_n_,_TT_.

**Table 2:** Characterization of diblock and triblock amphiphilic polymers based on PHPMA and PPO.

Polymer	Sample Code ^1^	*M*_n_ (SEC) ^2^ (g/mol)	*Ð*	% of PPO in Copolymer ^3^	*M*n (FFF) ^4^ (g/mol)	% of Micelles	*D*_h_, 37 °C (nm)	CMC^5^ (g L^−1^)
**P1**	DB-Boc	13.20	1.05	39.7	-	-	26.2	-
**P2**	DB-Boc-P	12.90	1.18	36.6	9.63 × 10^5^	88%	23.8	0.0503
**P3**	DB-H_2_O	14.11	1.09	35.1	1.20 × 10^6^	88%	26.6	0.0347
**P4**	DB-H_2_O-P	13.29	1.22	36.2	6.97 × 10^5^	76.5%	25.4	0.0558
**P5**	DB-TFA	13.72	1.11	40.4	1.15 × 10^6^	88%	27.4	0.0459
**P6**	TB-H_2_O-P	13.40	1.10	35.9	3.70 × 10^5^	45.3%	22.3	0.1307

^1^ DB: diblock, TB: triblock, Boc: Boc-protected hydrazides, H_2_O: hydrazides deprotected by water at high temperature, TFA: deprotection by acid hydrolysis, P: purified from free PPO and PHPMA copolymer. ^2^ determined by SEC, unimers, organic mobile phase 80% MeOH/20% acetic buffer (0.15 M, pH 6.5). ^3^ determined by NMR, 600 MHz, ^1^H, MeOH, 296 K from the ratio of HPMA and PPO. ^4^ determined by FFF, mobile phase water with NaN_3_, 10 kDa regenerated cellulose. ^5^ Critical micellar concentration: determined by fluorescence after 30 min of fluorescent dye addition (dye Nile Red) in PBS (10 mM, pH 7.4), concentration of dye 1 × 10^−6^ mol/L (λ_ex_ = 530 nm, λ_em_ = 610 nm).

**Table 3:** Description of the weight fraction of the loaded polymers P7–P14.

Sample Code	PPO/mg	Copolymer (P4)/mg	wt % of Loaded PPO *
**P4**	0	25	0
**P7**	1.35	25.55	5.02
**P8**	2.63	23.33	10.13
**P9**	4.51	25.54	15.01
**P10**	6.11	24.21	20.15
**P11**	8.31	24.75	25.14
**P12**	11.53	26.82	30.07
**P13**	13.52	24.97	35.13
**P14**	8.26	12.46	39.86

* wt % of PPO related to total weight of final loaded micelle.

**Table 4:** Characterization parameters of polymers P7–P14.

Sample Code	% of Total PPO in Copolymer ^1^	*M*_n_ (FFF) ^2^ (g/mol)	% of Micelle	*D*_h_, 37 °C (nm)	CMC ^3^ (g L^−1^)
**P4**	36.2	6.97 × 10^5^	76.5%	25.4	0.0558
**P7**	40.3	-	-	26.6	0.0585
**P8**	44.9	5.69 × 10^5^	75.3%	28.4	0.0524
**P9**	47.2	7.60 × 10^5^	80.2%	27.4	0.0339
**P10**	50.8	6.19 × 10^5^	78.7%	30.8	0.0506
**P11**	55.5	-	-	31.6	0.0443
**P12**	58.0	7.15 × 10^5^	71.6%	33.2	0.0438
**P13**	61.3	-	-	39.2	0.0422
**P14**	64.4	5.73 × 10^5^	70.2%	42.0	-

^1^ determined by NMR, 600 MHz, ^1^H, MeOH, 296 K from the ratio of HPMA and PPO. ^2^ determined by FFF, ratio of unimers and micelles, mobile phase water with NaN_3_, 10 kDa regenerated cellulose. ^3^ determined by fluorescence after 30 min of fluorescent dye addition (dye Nile Red) in PBS (10 mM, pH 7.4), concentration of dye 1 × 10^−6^ mol/L (λ_ex_ = 530 nm, λ_em_ = 610 nm).

## References

[1] Ulbrich K., Holá K., Šubr V., Bakandritsos A., Tuček J., Zbořil R. (2016). Targeted Drug Delivery with Polymers and Magnetic Nanoparticles: Covalent and Noncovalent Approaches, Release Control, and Clinical Studies. Chem. Rev..

[2] Markman J.L., Rekechenetskiy A., Holler E., Ljubimova J.Y. (2013). Nanomedicine therapeutic approaches to overcome cancer drug resistance. Adv. Drug Deliv. Rev..

[3] Szakács G., Paterson J.K., Ludwig J.A., Booth-Genthe C., Gottesman M.M. (2006). Targeting multidrug resistance in cancer. Nat. Rev. Drug Discov..

[4] Callaghan R., Luk F., Bebawy M. (2014). Inhibition of the multidrug resistance P-glycoprotein: Time for a change of strategy?. Drug Metab. Dispos..

[5] Wang Z., Chen Y., Liang H., Bender A., Glen R.C., Yan A. (2011). P-glycoprotein substrate models using support vector machines based on a comprehensive data set. J. Chem. Inf. Model..

[6] Chen L., Li Y., Yu H., Zhang L., Hou T. (2012). Computational models for predicting substrates or inhibitors of P-glycoprotein. Drug Discov. Today.

[7] Koziolová E., Janoušková O., Cuchalová L., Hvězdová Z., Hraběta J., Eckschlager T., Sivák L., Ulbrich K., Etrych T., Šubr V. (2016). Overcoming multidrug resistance in Dox-resistant neuroblastoma cell lines via treatment with HPMA copolymer conjugates containing anthracyclines and P-gp inhibitors. J. Control. Release.

[8] Šubr V., Sivák L., Koziolová E., Braunová A., Pechar M., Strohalm J., Kabešová M., Říhová B., Ulbrich K., Kovář M. (2014). Synthesis of Poly[*N*-(2-hydroxypropyl)methacrylamide] conjugates of inhibitors of the ABC transporter that overcome multidrug resistance in doxorubicin-resistant P388 cells in vitro. Biomacromolecules.

[9] Sivak L., Subr V., Tomala J., Rihova B., Strohalm J., Etrych T., Kovar M. (2017). Overcoming multidrug resistance via simultaneous delivery of cytostatic drug and P-glycoprotein inhibitor to cancer cells by HPMA copolymer conjugate. Biomaterials.

[10] Alakhova D.Y., Kabanov A.V. (2014). Pluronics and MDR reversal: An update. Mol. Pharm..

[11] Batrakova E.V., Kabanov A.V. (2008). Pluronic block copolymers: Evolution of drug delivery concept from inert nanocarriers to biological response modifiers. J. Control. Release.

[12] Kabanov A.V., Batrakova E.V., Alakhov V.Y. (2002). Pluronic^®^ block copolymers for overcoming drug resistance in cancer. Adv. Drug Deliv. Rev..

[13] Pitto-Barry A., Barry N.P.E. (2014). Pluronic^®^ block-copolymers in medicine: From chemical and biological versatility to rationalisation and clinical advances. Polym. Chem..

[14] Kabanov A.V., Zhu J., Kwon G.S. (2005). Pluronic Block Copolymers for Drug and Gene Delivery. Polymeric Drug Delivery Systems, Series: Drugs and the Pharmaceutical Sciences, Volume 148.

[15] Raval A., Pillai S.A., Bahadur A., Bahadur P. (2017). Systematic characterization of Pluronic^®^ micelles and their application for solubilization and in vitro release of some hydrophobic anticancer drugs. J. Mol. Liq..

[16] Batrakova E.V., Bronich T.K., Vetro J.A., Kabanov A.V. (2006). Polymer Micelles as Drug Carriers. Nanoparticulates as Drug Carriers.

[17] Fang J., Nakamura H., Maeda H. (2011). The EPR effect: Unique features of tumor blood vessels for drug delivery, factors involved, and limitations and augmentation of the effect. Adv. Drug Deliv. Rev..

[18] Maeda H. (2001). The enhanced permeability and retention (EPR) effect in tumor vasculature: The key role of tumor-selective macromolecular drug targeting. Adv. Enzym. Regul..

[19] Maeda H., Wu J., Sawa T., Matsumura Y., Hori K. (2000). Tumor vascular permeability and the EPR effect in macromolecular therapeutics: A review. J. Control. Release.

[20] Maeda H., Sawa T., Konno T. (2001). Mechanism of tumor-targeted delivery of macromolecular drugs, including the EPR effect in solid tumor and clinical overview of the prototype polymeric drug SMANCS. J. Control. Release.

[21] Braunová A., Kostka L., Sivák L., Cuchalová L., Hvězdová Z., Laga R., Filippov S., Černoch P., Pechar M., Janoušková O. (2017). Tumor-targeted micelle-forming block copolymers for overcoming of multidrug resistance. J. Control. Release.

[22] Nishiyama N., Matsumura Y., Kataoka K. (2016). Development of polymeric micelles for targeting intractable cancers. Cancer Sci..

[23] Cabral H., Kataoka K. (2014). Progress of drug-loaded polymeric micelles into clinical studies. J. Control. Release.

[24] Kataoka K., Harada A., Nagasaki Y. (2001). Block copolymer micelles for drug delivery: Design, characterization and biological significance. Adv. Drug Deliv. Rev..

[25] Kataoka K., Matsumoto T., Yokoyama M., Okano T., Sakurai Y., Fukushima S., Okamoto K., Kwon G.S. (2000). Doxorubicin-loaded poly(ethylene glycol)-poly(β-benzyl-l-aspartate) copolymer micelles: Their pharmaceutical characteristics and biological significance. J. Control. Release.

[26] Dongin K., Eun Seong L., Kyung Taek O., Zhong G.G., You Han B. (2009). Doxorubicin-Loaded Polymeric Micelle Overcomes Multidrug Resistance of Cancer by Double-Targeting Folate Receptor and Early Endosomal pH. Small.

[27] Deshmukh A.S., Chauhan P.N., Noolvi M.N., Chaturvedi K., Ganguly K., Shukla S.S., Nadagouda M.N., Aminabhavi T.M. (2017). Polymeric micelles: Basic research to clinical practice. Int. J. Pharm..

[28] Chytil P., Etrych T., Kříž J., Šubr V., Ulbrich K. (2010). *N*-(2-Hydroxypropyl)methacrylamide-based polymer conjugates with pH-controlled activation of doxorubicin for cell-specific or passive tumour targeting. Synthesis by RAFT polymerisation and physicochemical characterisation. Eur. J. Pharm. Sci..

[29] Ulbrich K., Etrych T., Chytil P., Jelínková M., Říhová B. (2004). Antibody-targeted Polymer–doxorubicin Conjugates with pH-controlled Activation. J. Drug Target..

[30] Šubr V., Koňák Č., Laga R., Ulbrich K. (2006). Coating of DNA/poly(l-lysine) complexes by covalent attachment of poly[*N*-(2-hydroxypropyl)methacrylamide]. Biomacromolecules.

[31] Koziolová E., Kostka L., Kotrchová L., Šubr V., Konefal R., Nottelet B., Etrych T. (2018). *N*-(2-Hydroxypropyl)methacrylamide-Based Linear, Diblock, and Starlike Polymer Drug Carriers: Advanced Process for Their Simple Production. Biomacromolecules.

[32] Perrier S., Takolpuckdee P., Mars C.A. (2005). Reversible addition-fragmentation chain transfer polymerization: End group modification for functionalized polymers and chain transfer agent recovery. Macromolecules.

[33] Zinelaabidine C., Souad O., Zoubir J., Malika B., Nour-Eddine A. (2012). A Simple and Efficient Green Method for the Deprotection of *N*-Boc in Various Structurally Diverse Amines under Water-mediated Catalyst-free Conditions. Int. J. Chem..

[34] Trousil J., Syrová Z., Dal N.J.K., Rak D., Konefal R., Pavlova E., Matějková J., Cmarko D., Kubíčková P., Pavliš O. (2019). Rifampicin Nanoformulation Enhances Treatment of Tuberculosis in Zebrafish. Biomacromolecules.

[35] Kolouchova K., Sedlacek O., Jirak D., Babuka D., Blahut J., Kotek J., Vit M., Trousil J., Konefał R., Janouskova O. (2018). Self-Assembled Thermoresponsive Polymeric Nanogels for 19F MR Imaging. Biomacromolecules.

[36] Von Richter O., Glavinas H., Krajcsi P., Liehner S., Siewert B., Zech K. (2009). A novel screening strategy to identify ABCB1 substrates and inhibitors. Naunyn. Schmiedeberg’s Arch. Pharmacol..

[37] Allen C., Maysinger D., Eisenberg A. (1999). Nano-engineering block copolymer aggregates for drug delivery. Colloids Surfaces B Biointerfaces.

[38] Nagarajan R., Ganesh K. (1989). 1989-Nagarajan. J. Chem. Phys..

[39] Klepac D., Kostková H., Petrova S., Chytil P., Etrych T., Kereïche S., Raška I., Weitz D.A., Filippov S.K. (2018). Interaction of spin-labeled HPMA-based nanoparticles with human blood plasma proteins-the introduction of protein-corona-free polymer nanomedicine. Nanoscale.

[40] Tyrrell Z.L., Shen Y., Radosz M. (2010). Fabrication of micellar nanoparticles for drug delivery through the self-assembly of block copolymers. Prog. Polym. Sci..

[41] Lammers T., Kühnlein R., Kissel M., Subr V., Etrych T., Pola R., Pechar M., Ulbrich K., Storm G., Huber P. (2005). Effect of physicochemical modification on the biodistribution and tumor accumulation of HPMA copolymers. J. Control. Release.

[42] Sajid M.S., Jabeen F., Hussain D., Ashiq M.N., Najam-ul-Haq M. (2017). Hydrazide-functionalized affinity on conventional support materials for glycopeptide enrichment. Anal. Bioanal. Chem..

[43] Roth J. (2002). Protein *N*-glycosylation along the Secretory Pathway: Relationship to organelle topography and function, protein quality control, and cell interactions. Chem. Rev..

